# Noise-Robust Wafer Map Defect Classification via CNN-ESN Hybrid Architecture

**DOI:** 10.3390/mi17030309

**Published:** 2026-02-28

**Authors:** Hayeon Choi, Dasom Im, Sangeun Oh, Jonghwan Lee

**Affiliations:** Department of System Semiconductor Engineering, Sangmyung University, Cheonan 31066, Republic of Korea

**Keywords:** wafer map, defect classification, convolutional neural network, echo state network, robustness analysis, reservoir computing, semiconductor manufacturing

## Abstract

Wafer map defect classification plays a critical role in yield monitoring and root-cause analysis in semiconductor manufacturing. Although recent convolutional neural network (CNN)-based approaches have achieved high classification accuracy, most existing models are evaluated primarily on clean datasets and remain vulnerable to unseen perturbations and representation-level variability at test time. In this paper, we propose a hybrid CNN–echo state network (ESN) architecture that integrates spatial feature extraction with sequential aggregation to enhance robustness under input perturbations. The CNN backbone extracts two-dimensional feature maps, which are converted into ordered sequences using a multidirectional scanline strategy and processed by an ESN reservoir. The resulting sequential representations are combined with CNN features through a class-specific adaptive fusion mechanism. Using the defect-only eight-class version of the WM-811K dataset, we systematically evaluate robustness under multiple perturbation scenarios, with particular focus on the clean train/noisy test (CT-NT) setting. To ensure a controlled robustness evaluation aligned with the binary nature of wafer map data, we introduce binary-consistent die-flip perturbations and additionally employ additive Gaussian perturbations as a representation-level stress test. Under clean-data conditions, the proposed model showed a 0.61 pp improvement in test accuracy compared to the ResNet34-based CNN, with notably larger gains for rare classes and defect types exhibiting strong structural patterns. In the clean train/noisy test scenario, where the model was trained on clean wafer map data and evaluated under controlled test-time perturbations, the accuracy of the CNN baseline dropped to 77.59% at σ = 0.10, whereas the proposed hybrid model maintained an accuracy of 87.30%, resulting in an absolute improvement of 9.71 pp. Per-class analysis reveals that the robustness gain is class-dependent, with pronounced improvements for defect types exhibiting clear and repetitive structural patterns, such as Loc and Edge-Ring. Further mechanistic analysis demonstrates that the robustness improvement arises from enhanced representation stability and bounded reservoir dynamics, rather than from changes in CNN feature extraction or training regularization. These results demonstrate that the proposed CNN-ESN hybrid architecture provides meaningful advantages in terms of robustness under noisy evaluation conditions without requiring noise-aware training or prior knowledge of perturbation characteristics.

## 1. Introduction

In semiconductor manufacturing processes, wafer map defect patterns directly affect the product yield and quality, making automated techniques for early defect classification critical for detecting process anomalies and improving manufacturing efficiency. In recent years, automatic classification techniques based on convolutional neural networks (CNNs) have been actively studied in wafer map defect classification research. Various architectures, including the Visual Geometry Group (VGG) network, ResNet, and EfficientNet, have been applied to datasets similar to WM-811K, with reported accuracies exceeding 90% [1]. Attempts have been made to mitigate class imbalance through data augmentation [2]. However, most existing studies primarily evaluate classification performance under refined (clean) data conditions and do not explicitly examine model behavior under distribution shifts or unseen perturbations at test time. Existing CNN-based wafer map defect classification methods primarily focus on static spatial patterns in two-dimensional images, and thus ordered spatial dependencies and progressive structural characteristics associated with certain defect types are not explicitly modeled. Furthermore, CNN-based deep learning models are highly dependent on the distribution of training data. It has been reported that their generalization performance for minority classes degrades rapidly in imbalanced data environments dominated by majority classes. A related review also reported that existing deep-learning-based techniques still suffer from imbalance in data, emergence of new defect patterns, and limited robustness under varying process conditions [3]. Meanwhile, prior studies in circuit design and electronic design automation have shown that deep learning models can be highly sensitive to small input perturbations or adversarial noise [4,5].

In the context of wafer map analysis, such perturbations should be interpreted as abstract representation-level stress rather than direct physical process noise, as wafer map data are inherently discrete and binary. Nevertheless, variations in inspection conditions, labeling uncertainty, or data acquisition pipelines can introduce test-time perturbations that are not observed during training. Under such conditions, achieving high accuracy on clean datasets alone is insufficient to guarantee stable deployment. Therefore, there is a need for model architectures that not only achieve high nominal accuracy but also maintain representation stability under unseen perturbations.

To address these limitations, this study introduces a hybrid architecture that integrates CNNs, which excel at learning static spatial representations, with reservoir computing, which is effective for sequential aggregation and dynamic representation. In this study, feature maps extracted by CNNs from wafer maps are provided as inputs to an echo state network (ESN). Specifically, two-dimensional feature maps were segmented into ordered sequences using a multidirectional scanline strategy and then fed into the ESN reservoir. Subsequently, patterns affected by input perturbations were transformed into ordered sequences and aggregated as reservoir states. By combining the static spatial pattern representation learned by the CNN with the sequence-driven dynamic representations learned by the ESN, the proposed CNN-ESN hybrid architecture is expected to provide more stable feature representations under test-time perturbations than CNN-only models. By introducing a multidirection scanline structure, defect patterns on wafer maps were projected onto ordered sequences from multiple directions, forming a richer sequential representation space compared to that corresponding to single-direction sequences, increasing model sensitivity to mixed patterns and structural pattern variations.

To evaluate the effectiveness of the proposed CNN-ESN hybrid architecture under clean train/noisy test (CT-NT) conditions, this study established four evaluation scenarios combining different noise intensities and injection stages (CT-CT, NT-CT, NT-NT, and CT-NT) and systematically analyzed performance changes under each condition. In particular, we focused on the CT-NT scenario, where perturbations are introduced only during the testing phase. We demonstrated that the hybrid structure exhibited a much more gradual performance degradation compared to conventional CNN-based models. Thus, this article introduces a CNN-ESN-based sequential representation framework that jointly reflects static spatial patterns of wafer map defects and ordered spatial variation induced by test-time perturbations, enhances robustness without noise-aware training, and improves performance generalization for rare classes and composite patterns through class-weight-based adaptive fusion and the multidirectional scanline structure.

## 2. Designing and Training a CNN-Based Baseline Model

### 2.1. WM-811K Dataset and Data Preprocessing (Data Imbalance and Preprocessing)

**Figure 1:**
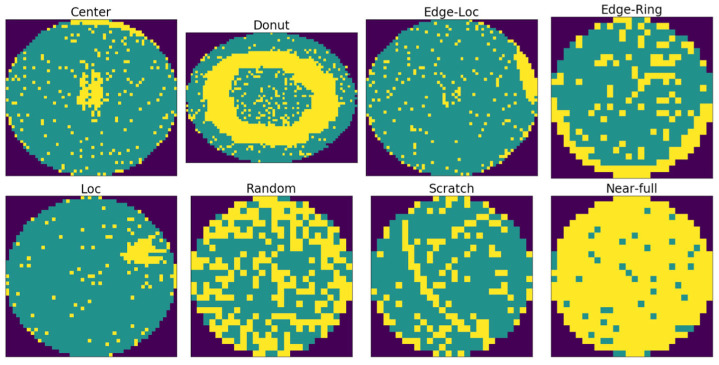
Defect Pattern Examples from the WM-811K Dataset. Yellow represents defect data, green denotes normal (pass) data, and the dark outer areas are the background.

This study used an LSWMD.pkl file, a Kaggle-distributed version of Wafer Map-811K (WM-811K) dataset, the most widely used public dataset for wafer map defect classification [6]. This version reconstructs the hierarchical dictionary structure of the original data into a pandas DataFrame format, providing a refined structure ready for large-scale deep learning experiments. Among the 811,457 wafer maps collected from semiconductor manufacturing data, 172,950 samples are labeled by domain experts into nine defect types: Center, Donut, Edge-Loc, Edge-Ring, Loc, Random, Scratch, Near-full, and None. However, this labeled data contains a severe class imbalance. Among the 172,950 samples, the normal-defect-pattern class None comprises 147,431 samples (85.2%), while the actual defect-pattern class accounts for only 14.8%. For this structure, a model that simply predicts the None pattern can achieve an accuracy exceeding 85%, making it difficult to fairly assess model performance. In practical inspection settings, most wafers are normal, so the main objective is not normality detection but accurate identification of defect types when defects occur. Therefore, following the standard approach adopted in previous WM-811K-based studies, the present study considered the classification task with only eight defect classes, excluding the None class. Representative examples of the eight defect patterns considered in this study are shown in Figure 1.

During data preprocessing, inconsistent label expressions were first normalized to unify the label scheme. Then, the None class was removed to construct a defect-only dataset consisting of 25,519 defect samples. Since wafer maps have inconsistent resolutions and measurement scales due to equipment and process differences, all samples were normalized to ensure a common input format. Missing values in inactive regions were replaced with 0 to ensure computational stability. To compensate for intensity differences between samples, min-max normalization was applied to each wafer map, unifying pixel values to the [0, 1] range. All wafer maps were then resized to a resolution of 64 × 64 pixels to preserve the spatial structure of defect patterns while ensuring high computational efficiency. This choice was based on previous research, which has revealed that compared to higher resolutions (128 × 128 or above), this resolution reduces memory usage by more than a factor of four while incurring only minimal performance loss [7]. The normalized samples were converted into the float32 single-precision format, making them suitable for deep neural network processing.

The resulting defect-only dataset was split into training (70%), validation (15%), and test (15%) sets using stratified sampling to maintain class proportions. In particular, for highly rare classes such as Near-full and Scratch, simple random partitioning poses a high risk of certain subsets likely lacking sufficient samples. Therefore, stratified splitting was employed to ensure a stable distribution across training, validation, and test sets, enabling reliable evaluation. However, even after this splitting, class imbalance of nearly 70:1 persisted between Edge-Ring (41.2%) and Near-full (0.6%) classes. This residual imbalance could have caused the model to overemphasize the majority class or fail to learn adequate representations for rare classes. Hence, class-weighted loss (weighted loss) based on the inverse class frequency was used [3]. The weight for each class i was defined as follows:

(1) $$W_{i}=\frac{N}{N_{i}\times C}$$

**Figure 2:**
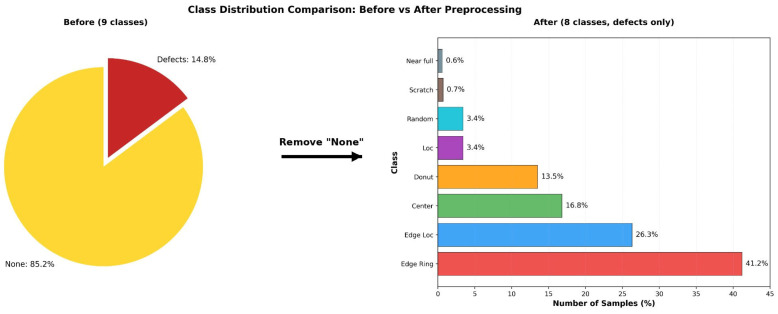
Class Distribution Before and After Data Preprocessing.

Here, N is the total number of training samples, $N_{i}$ is the number of training samples in class i, and C is the total number of classes (i.e., 8). These weights amplify the contributions of rare classes (e.g., Scratch and Near-full) while reducing the influence of majority classes (e.g., Edge-Ring), guiding the model toward balanced representation learning. In our experiments, the recall for rare classes, which was below 40% prior to weighting, improved to >70% after applying class weights. Thus, this weighting approach contributed to better recall for rare classes and ensured that the model responded to different defect patterns in a balanced manner. Figure 2 illustrates the change in class distribution before and after data preprocessing.

Although the original dataset exhibited extreme class imbalance, the defect-only dataset showed a more balanced form across the eight defect classes after data preprocessing. This data structure provided a foundation for CNN and hybrid CNN-ESN models to reliably learn the structural characteristics of different defect patterns while serving as an important basis for establishing a fairer and more controlled evaluation framework for defect classification. The final dataset was stored in the NumPy format. Key preprocessing settings, such as the defect-only class configuration, normalization method, data split ratios, and class distribution information, are provided alongside metadata files to ensure reproducibility and data integrity.

### 2.2. CNN Model Architecture and Components

**Figure 3:**
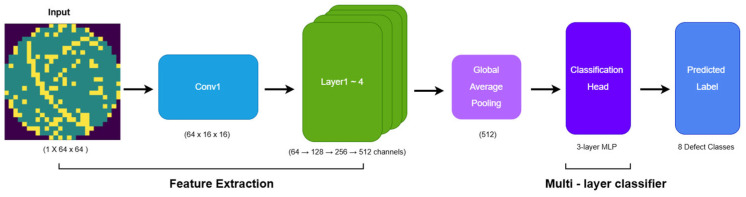
CNN Architecture.

The overall architecture of the CNN baseline model is illustrated in Figure 3. The baseline model fully inherits the structural advantages of ResNet-34. Residual blocks with skip connections alleviate gradient vanishing and provide stable representation learning for wafer defect data exhibiting both local irregularities and global structural variations [8]. To process single-channel wafer maps, the original 3-channel conv1 layer is replaced with a convolution layer with a kernel size of 7 × 7 (stride 2, padding 3), followed by Batch Normalization, ReLU, and MaxPooling [9,10]. The ResNet backbone (layer1–layer4) maintains its original residual structure, enabling robust learning against structural deformation and pattern variability. The final feature map, consisting of 512-channels, is converted into a 512-dimensional feature vector through Global Average Pooling, which extracts global statistics without increasing model parameters [11].

The classification head is implemented as a multi-stage MLP that gradually compresses the GAP output (512 → 256 → 128), where Batch Normalization and Dropout rates of 0.3 and 0.15 are applied to stabilize feature distribution and suppress overfitting under class imbalance. This CNN baseline preserves the original ResNet architecture without any external modules or ESN integration, serving as a fair and stable comparison reference to quantitatively validate the performance gain of the proposed hybrid CNN-ESN model.

## 3. CNN-ESN Hybrid Structure

### 3.1. Overview of the Overall Architecture

**Figure 4:**
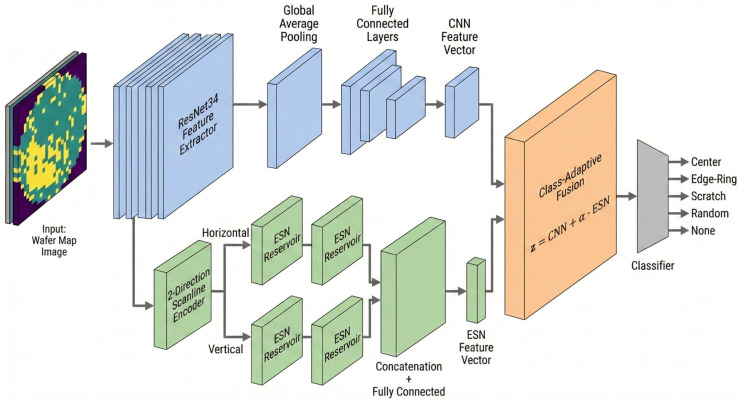
CNN-ESN Hybrid Model Neural Network.

The Conventional CNN-based wafer map defect classification models achieve high accuracy on clean datasets. However, prior studies have reported that these models remain sensitive to input perturbations and unseen test-time variability not seen during training [12,13,14]. Figure 4 shows the proposed hybrid CNN-ESN architecture designed to address these limitations, displaying the overall processing flow that integrates the spatial pattern representation capabilities of CNNs with the sequence-driven recurrent representation capability of ESNs. The input wafer map is transformed into an intermediate feature map F through a ResNet34-based CNN backbone. The feature map is then bifurcated into a CNN branch and an ESN branch. The CNN branch includes a CNN classification head that performs GAP and consists of an MLP, which effectively captures the global structure and fixed spatial patterns of defects. The ESN branch converts the 2-D feature map into sequential data by scanning it along the horizontal and vertical directions and then feeds the resulting sequences into the ESN.

This structure is designed to model the directional characteristics, progression, and structural variability of defect patterns from a sequential perspective. The representations from both branches are ultimately combined in an adaptive fusion module, which dynamically adjusts the relative contributions of the CNN and ESN branches according to the characteristics of each defect class. This architecture structurally addresses the noise vulnerability and directional pattern recognition limitations inherent in CNN-only models.

### 3.2. Overview of the ESN

**Figure 5:**
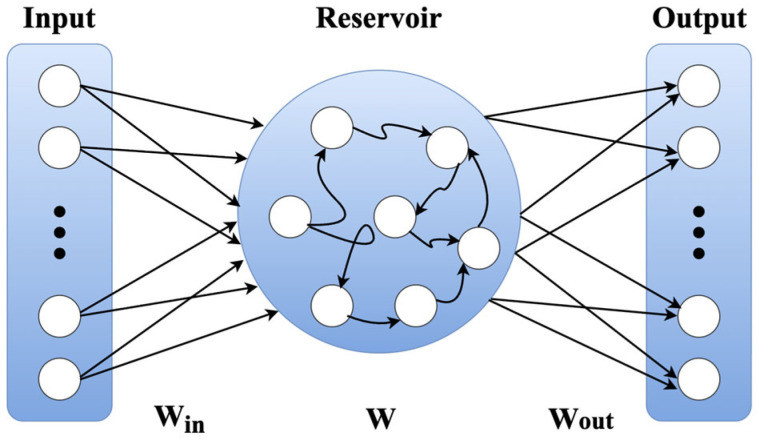
Basic structure of the ESN model.

ESNs are reservoir computing models that keep the internal recurrent weights W fixed rather than learning and varying them, and they model nonlinear dynamics by projecting the input sequence into a high-dimensional state space [15,16]. Figure 5 illustrates the basic structure of an ESN model, showing a typical flow where input sequences pass through a reservoir with fixed recurrent connections.

Subsequently, the sequences are linearly combined in the output layer to produce a prediction. The state update equation for the Leaky ESN used in this study is expressed as follows:

(2) $$h\left(t\right)=\left(1-\alpha \right)h\left(t-1\right)+\lambda tanh(W_{in}x\left(t\right)+Wh\left(t-1\right))$$

Here, the leaking rate λ controls the degree of past-state preservation, with a small λ value emphasizing long-term dependence and a large λ value emphasizing short-term changes.

Unlike a classical ESN, which trains only the output weights using ridge regression, the ESN in this study does not rely on ridge regression. Instead, the final reservoir state is passed to a trainable ESN head composed of a Fully Connected (FC) layer, Batch Normalization (BN), ReLU, and Dropout. Although the reservoir weights remain fixed, the CNN backbone and ESN output head are jointly optimized via backpropagation. This enables joint optimization of static spatial representations learned by the CNN and dynamic representations

### 3.3. Two-Direction Scanline-Based Sequence Encoding

**Figure 6:**
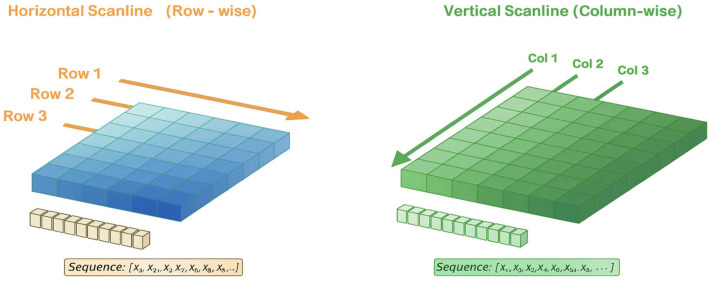
Two-Direction Scanline Encoding Strategy.

Wafer map defects often exhibit characteristics that propagate or progress in specific directions, which is difficult to fully capture with two-dimensional convolution applied at a single time step. For example, Scratch, Loc, and Edge-Loc patterns display clear directionality and localized spreading structures, while Donut and Edge-Ring patterns exhibit gradual spatial transitions [13,14]. To reflect these characteristics, the CNN feature map F is unfolded into scanline sequences along the horizontal (H) and vertical (V) directions, forming a two-direction ESN. Figure 6 shows the overall decomposition flow of the feature map into two orthogonal directions, converting each into a sequence, followed by the processing of each sequence with a separate ESN. The final outputs of the two ESNs are combined in parallel, providing a richer multi-directional sequential representation than that obtained from a single-direction ESN. As a result, this architecture improves the robustness of the learned feature representation and maintains stable classification performance, even in the presence of directional pattern variations, localized noise that corrupts local wafer regions and distorts local feature consistency, and structural distortions.

### 3.4. Noise Robustness of Reservoir Networks

Numerous studies have pointed out that deep learning models are highly sensitive to input noise, and CNNs, in particular, tend to exhibit abrupt output fluctuations even under subtle perturbations [15]. ESNs, however, exhibit structural properties that make them robust to input noise [17]. The tanh activation function of ESNs is insensitive to input changes in the saturation region and hence attenuates high-frequency noise. In addition, the recurrent structure diffuses the input signal across the entire reservoir state, leading to a sequence-level averaging effect that reduces the influence of localized noise. Furthermore, the scanline-based sequence representation helps dilute pixel-level perturbations through accumulated information along the sequence direction, leading to high overall stability. Due to these structural characteristics, the ESN branch shows a comparatively smaller degree of performance degradation than the CNN under the clean train/noise test (CT-NT) condition, a result that is later confirmed in the experimental analysis.

### 3.5. Class-Specific Adaptive Fusion Strategy

Since the wafer map defect dataset exhibits large differences in sample counts across classes, class imbalance arises easily during training [13,14]. To mitigate this, we used a class-weighted cross-entropy loss that took into account the sample proportion of each class. The class weight was defined as a function of the number of samples $n_{c}$ in each class:

(3) $$L_{cls}=\;-\sum_{c=1}^{K}w_{c}y_{c}\log\left({\hat{y}}_{c}\right),\;\;w_{c}=\;\frac{N}{K\;n_{c}}$$

Furthermore, because defect types show differences in their degree of sequential dependence and structural characteristics, we designed an adaptive fusion strategy that dynamically adjusts the contribution of the ESN branch to the model output. The fusion weight α was calculated as follows, based on the CNN’s softmax response $p_{cnn,c}\;$ and the class-specific ESN importance $\omega_{c}$:

(4) $$\alpha =\sum_{c=1}^{K}p_{cnn,c}\omega_{c}$$

The final fused representation is defined as a residual fusion form, where the CNN feature $h_{cnn}$ serves as the base representation and the ESN feature $h_{esn}$ is added as a class-dependent residual scaled by α:

(5) $${\;h}_{fusion}=h_{cnn}+\alpha h_{esn}$$

Classes with strong sequential or progressive structural characteristics, such as Scratch, Loc, and Donut, were assigned higher $\omega_{c}$ values, resulting in larger α and a stronger contribution from the ESN branch. In contrast, classes with predominantly static structural characteristics, such as Center and Random, were assigned lower values, allowing the CNN representation to remain dominant. This residual fusion design enables more balanced and class-adaptive representation learning by reflecting the pattern-specific properties of each defect class. Importantly, this adaptive fusion strategy is designed to mitigate class-specific confusion patterns rather than to maximize per-class accuracy independently, as different defect classes benefit differently from sequential information.

## 4. Experimental Results

### 4.1. Comparison with State-of-the-Art Methods

To contextualize the performance of the hybrid CNN-ESN model, we compared it with state-of-the-art methods using the WM-811K dataset. As summarized in Table 1, prior studies mainly report test accuracy under clean, unperturbed wafer maps, with performance typically ranging from about 90% to 98%. These works focus on classification accuracy under nominal conditions and do not explicitly analyze performance stability under inspection-induced variation or distribution shift.

In contrast, this study evaluates performance not only on clean data but also under controlled input perturbations, where models are trained on clean data and evaluated on noisy test data (CT-NT), in order to examine robustness to inspection-induced representation shift not observed during training. The added perturbations are primarily used as abstract stress tests of representation stability rather than as physical noise models. Therefore, the main contribution of this work is not to maximize clean-condition accuracy, but to provide a systematic evaluation and improvement of robustness under simulated process-induced variation. Accordingly, direct accuracy comparisons under clean conditions should be interpreted cautiously, as prior studies focused on nominal-condition performance and did not evaluate robustness or sensitivity to distribution shift. While the primary perturbations are introduced as abstract stress tests of representation stability, later sections additionally consider binary-consistent perturbations and noise-injected training scenarios to provide complementary robustness analysis.

### 4.2. Experimental Setup and Evaluation Protocol

#### 4.2.1. Experimental Setup and Hyperparameters

The performance evaluation of the proposed hybrid CNN-ESN model was performed using the PyTorch (version 2.9.0) with Python 3.11 under a CUDA 12-enabled enviornment. As described in Section 2.1, data preprocessing and training/validation/test stratified splitting were performed to construct the WM-811K defect-only dataset. Training was performed for up to 80 epochs, with a fixed batch size of 64. For optimization, AdamW (learning rate = $5\times 10^{-4}$; weight decay = $1\times 10^{-4}$) was used. The learning rate scheduler was cosine annealing with warm restarts ($T_{0}=20$), and early stopping based on validation accuracy was applied to prevent overfitting. All experiments were independently repeated using five random seeds, and the mean and standard deviation values are reported. Key hyperparameters for the ESN reservoir were determined by grid search based on the validation set performance. Independent ESN reservoirs were used for the horizontal and vertical directions, with a reservoir size of 300, spectral radius of 0.99, leakage rate of 0.9, and density of 0.15. The class-specific adaptive fusion coefficients $\omega_{c}$ discussed in Section 3.5 were designed to control the relative contribution of the ESN-based sequential representation to the final class prediction, with higher $\omega_{c}$ values assigned to defect classes exhibiting stronger directional or progressive patterns. These fusion weights were empirically determined based on validation performance and consistent per-class confusion patterns. The specific values used in the experiments are summarized in Table A1 (Appendix A).

#### 4.2.2. Evaluation Metrics and Statistical Testing

Model performance was evaluated using overall accuracy, per-class accuracy, and macro F1 scores. When the number of test samples is N, the overall accuracy is defined as follows:

(6) $$Accuracy=\frac{1}{N}\sum_{k=1}^{N}1({\hat{y}}_{k}=y_{k})$$

Here, ${\hat{y}}_{k}$ is the class predicted by the model, $y_{k}$ is the actual label, and 1(⋅) is the indicator function.

Per-class accuracy was calculated using the same formula independently for samples from each class. We also used the macro F1 score to account for class imbalance. The macro F1 score is obtained by computing the F1 score for each class and then taking their arithmetic mean:

(7) $$\mathrm{macro-F1}=\frac{1}{c}\sum_{c=1}^{c}{F1}_{c}$$

Here, C is the number of classes (i.e., 7) and ${F1}_{c}$ is the F1 score for class C. To test whether the performance difference between the CNN baseline and the hybrid CNN-ESN model was statistically significant, we conducted a paired t-test using the distribution of accuracies obtained from five independent runs with different random seeds. The null hypothesis $H_{0}$ was “the mean accuracy of the two models is equal.” A *p*-value of <0.05 indicates a statistically significant difference. We also used Cohen’s d to quantitatively assess the effect size:

(8) $$d=\frac{\mu_{hybrid}-\mu_{baseline}}{\sigma_{pooled}}$$

Here, $\mu_{hybrid}$ and $\mu_{baseline}$ are the mean accuracies of the models and $\sigma_{pooled}$ is the pooled standard deviation. In general, d > 0.8 is considered to represent a large effect. Statistical significance testing is reported for the CT-CT baseline comparison, whereas robustness under perturbation is evaluated based on consistent performance trends across noise levels.

### 4.3. Clean-Data Performance

#### 4.3.1. Overall Performance Comparison

We first compare the classification performance of the CNN baseline and the proposed hybrid CNN-ESN model under the clean train/clean test (CT-CT) setting, where no perturbation is applied during either training or evaluation. All experiments were repeated independently using five different random seeds. As summarized in Table 2, the CNN baseline achieved a mean test accuracy of 94.13% ± 0.25%, while the proposed hybrid CNN-ESN model achieved 94.74% ± 0.24%, corresponding to an absolute improvement of 0.61 pp. A paired *t*-test yields a *p*-value of 0.036, indicating that the observed difference is statistically significant despite the relatively small absolute gain under clean conditions. The corresponding Cohen’s d value of 2.49 reflects a large effect size, which should be interpreted in the context of repeated runs with low variance under clean-data conditions, rather than from a large absolute performance difference. These results indicate that incorporating the ESN branch does not degrade clean-data performance and yields a modest but consistent improvement over the CNN-only baseline.

#### 4.3.2. Per-Class Performance Analysis

To further analyze class-wise behavior under clean conditions, Table 3 reports per-class accuracy for both models in the CT-CT scenario. The hybrid model shows larger relative gains for classes with limited sample sizes or stronger structural characteristics. In particular, improvements of +4.22 pp for Near-full, +1.86 pp for Loc, and +1.24 pp for Center are observed. These classes are characterized by distinctive spatial layouts or directional patterns, where sequential aggregation can provide complementary structural cues. In contrast, for classes such as Donut and Random, where the CNN baseline already achieves stable and high accuracy, no further improvement is observed. This suggests that the ESN branch primarily contributes when additional structural regularization is beneficial, rather than uniformly increasing accuracy across all classes.

#### 4.3.3. Confusion Matrix Analysis

**Figure 7:**
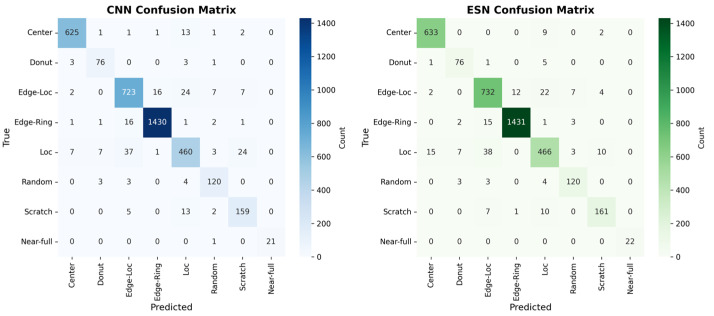
Confusion Matrices for the CNN baseline and Hybrid CNN-ESN model in the CT-CT Scenario.

Figure 7 shows the confusion matrices of the CNN baseline and the hybrid CNN-ESN model in the CT-CT scenario, providing a qualitative view of prediction tendencies under clean-data conditions. Overall, both models exhibit similar confusion patterns, reflecting their comparable overall accuracy. Consistent with the per-class accuracy trends reported in Table 3, the hybrid model exhibits slightly reduced misclassification for several structurally directional defect classes. For example, the improvements observed for Edge-Loc (82.70% → 83.86%) and Loc (85.34% → 87.20%) correspond to a modest reduction in confusion with spatially similar classes such as Edge-Ring and Center. For the Donut class, although the per-class accuracy remains unchanged, the confusion matrix reveals a small redistribution of misclassified samples across neighboring categories. This suggests that the adaptive fusion mechanism subtly reshapes decision boundaries without necessarily increasing the accuracy for classes that are already well classified under clean conditions. Taken together, the confusion matrix analysis complements the quantitative results by qualitatively illustrating more stable class separation for structurally related defect patterns while preserving overall prediction behavior in the clean-data setting.

### 4.4. Robustness Evaluation Under Perturbations

#### 4.4.1. Binary-Consistent Perturbation Robustness

To evaluate robustness under perturbations that are consistent with the binary nature of wafer map data, we introduce a die-flip perturbation strategy and adopt it as the primary robustness evaluation setting. Unlike additive Gaussian noise, which produces intermediate pixel values, the die-flip perturbation strictly preserves the binary domain by randomly flipping the state of individual dies from pass to fail or vice versa. This perturbation does not model physical process noise; instead, it introduces controlled inspection-level uncertainty that may arise from die-level mislabeling or annotation variability in wafer map representations [24]. The purpose of this perturbation is not to simulate physical manufacturing noise, but to provide a binary-consistent robustness benchmark that allows systematic analysis of model stability under controlled discrete perturbations.

Formally, given a binary wafer map $x\;\epsilon \;{\left\{0,1\right\}}^{\left\{H\times W\right\}}$, each die value is independently flipped with probability p. In this study, we consider p ϵ {0.001,0.005,0.01,0.02}, covering a range of perturbation intensities from mild to severe binary-consistent uncertainty. All models are trained exclusively on clean binary wafer maps and evaluated under the clean train/noisy test (CT-NT) setting, ensuring that robustness is assessed solely based on the model’s ability to tolerate unseen perturbations at test time.

Table 4 summarizes the classification accuracy of the CNN baseline and the proposed hybrid CNN-ESN model under increasing die-flip probabilities. Although the absolute performance gap under die-flip perturbation is smaller than that observed under Gaussian stress tests, the hybrid CNN-ESN model consistently outperforms the CNN baseline across all flip probabilities. Moreover, as the perturbation intensity increases, the accuracy gap between the two models gradually widens, indicating that the hybrid architecture degrades more gracefully under binary-consistent perturbations. 

These results demonstrate that the robustness of the proposed hybrid CNN-ESN model is not limited to representation-level stress tests, but also extends to binary-consistent perturbations that preserve the discrete nature of wafer map data. The consistent advantage observed across all die-flip levels suggests that ESN-based sequential aggregation contributes to stabilizing spatial representations even when local binary disruptions are present.

#### 4.4.2. Representation-Level Stress Test

To further analyze robustness beyond binary-consistent perturbations, we additionally evaluate the models under additive Gaussian perturbations as a representation-level stress test. Unlike the die-flip perturbation introduced in Section 4.4.1, Gaussian noise does not preserve the binary nature of wafer map data and therefore does not correspond to a realistic inspection or labeling process. Instead, it is employed here as an abstract mechanism to probe the stability of learned feature representations under continuous-valued perturbations.

Formally, Gaussian noise is injected into a normalized input representation $x\epsilon \;{\left\{0,1\right\}}^{\left\{1\;\times H\times W\right\}}$ as follows:

(9) $$\tilde{x}=x+\varepsilon ,\varepsilon \;\sim N\left(0,\sigma^{2}\right)$$

where σ controls the magnitude of perturbation. In this study, we consider σ∈{0.05,0.08,0.10} covering a range from mild to severe representation distortion. All models are trained exclusively on clean binary wafer maps and evaluated under the clean train/noisy test (CT-NT) setting, ensuring that robustness reflects tolerance to unseen representation shifts rather than adaptation through noise-aware training.

Table 5 summarizes the classification accuracy of the CNN baseline and the proposed hybrid CNN-ESN model under increasing Gaussian noise levels. As the perturbation magnitude increases, the CNN baseline exhibits a rapid degradation in performance. In contrast, the hybrid CNN-ESN model demonstrates substantially slower performance decay, resulting in an increasingly large accuracy gap at higher noise levels. Notably, under the most severe perturbation (σ = 0.10), the hybrid model outperforms the CNN baseline by a margin of 9.71 percentage points.

**Figure 8:**
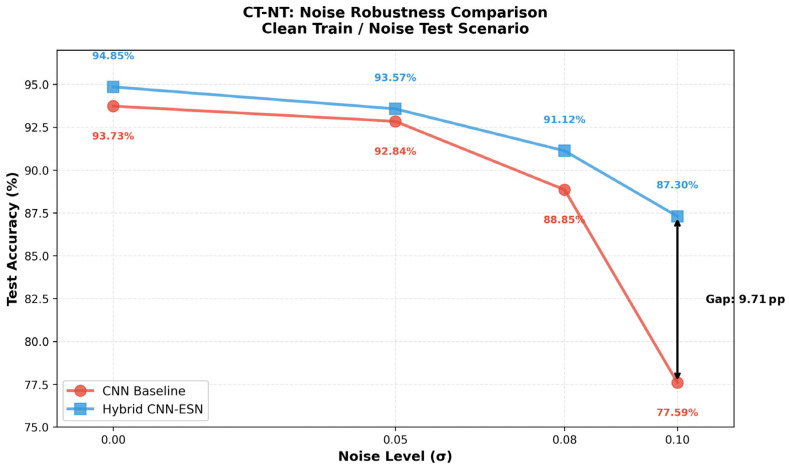
Noise Robustness Curves in the CT-NT scenario comparing the CNN baseline and the Hybrid CNN-ESN model.

These trends are further visualized in Figure 8, which shows the noise robustness curves of both models under the CT-NT setting. While the performance gap under clean conditions is marginal (0.61 pp), it widens sharply as noise intensity increases, highlighting the superior degradation behavior of the hybrid architecture under representation-level stress.

Although Gaussian perturbation does not reflect a physically plausible noise process for wafer map data, these results provide complementary insight into the internal stability of the learned representations. The observed robustness suggests that the ESN-based sequential aggregation contributes to smoothing local perturbations and stabilizing intermediate feature dynamics, even under continuous-valued stress conditions that violate the original binary input assumption. Importantly, these findings should be interpreted strictly as evidence of representation-level robustness, rather than as a claim of improved performance under realistic manufacturing noise. Accordingly, the Gaussian perturbation analysis is used solely to support the mechanistic analysis of feature stability presented in Section 4.4.

Detailed per-class robustness results under high noise conditions are provided in Appendix A.3.

### 4.5. Mechanistic Analysis of Noise Robustness

The robustness trends observed in the CT-NT scenario can be explained by the design principles of the proposed hybrid architecture described in Section 3.

Specifically, the multidirectional scanline transformation induces a sequence-based smoothing effect that disperses localized pixel-level perturbations across the ordered sequence dimension.

In addition, the tanh nonlinearity of the ESN reservoir naturally attenuates high-frequency disturbances through saturation, while the class-specific adaptive fusion strategy adjusts the contribution of the ESN branch according to the structural characteristics of each defect type.

To further substantiate this interpretation beyond classification accuracy, we analyze the stability of internal representations and decision-level behavior under controlled perturbations.

#### 4.5.1. Representation Stability Under Perturbations

**Figure 9:**
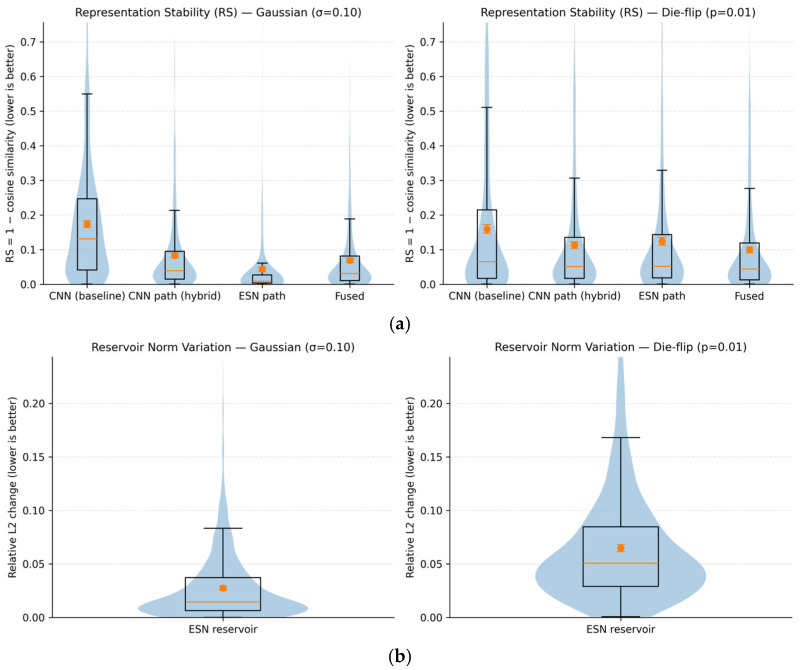

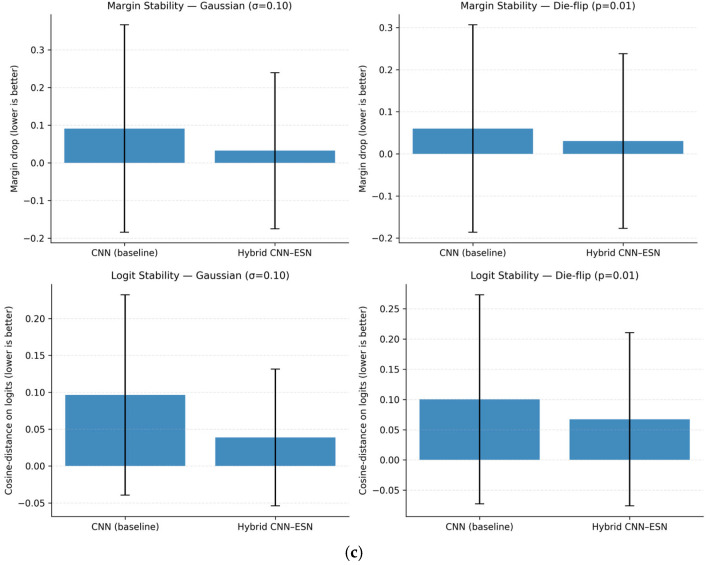
Representation and decision stability under Gaussian and binary-consistent perturbations: (**a**) Representation stability (RS) distributions; (**b**) Relative L2 variation in ESN reservoir states; (**c**) Margin drop and logit change comparison.

To verify robustness beyond classification accuracy, we analyze how internal feature representations change when controlled perturbations are applied to the same wafer map. We examine four representations: the output of the CNN baseline (CNN), the CNN branch output of the hybrid CNN-ESN model (CNN path), the ESN-aggregated representation (ESN path), and the final fused representation used for classification (Fused). Note that the CNN baseline and the CNN path share the same backbone architecture and training protocol; the CNN path corresponds to the intermediate CNN features within the hybrid CNN-ESN model before ESN-based aggregation. Representation stability (RS) is defined as the cosine distance between features from clean and perturbed inputs:

(10) $$RS=1-cos(r_{clean},\;r_{perturbed})$$

where smaller values indicate higher robustness. As shown in Figure 9a, under both Gaussian perturbations (σ = 0.10) and binary-consistent die-flip perturbations (*p* = 0.01), the ESN path and fused representations consistently exhibit lower RS values than both the CNN baseline and the CNN path representations. This observation confirms that the robustness gain is not attributable to differences in CNN feature extraction or training strength, but arises specifically from the ESN-based sequential aggregation and the subsequent fusion mechanism. Importantly, the CNN path of the hybrid model shows RS behavior comparable to that of the CNN baseline, suggesting that the observed stability gain does not arise from differences in CNN training, but from the subsequent ESN-based aggregation and fusion.

#### 4.5.2. Reservoir Dynamics and Decision-Level Stability

To further analyze how ESN aggregation stabilizes representations, we examine the dynamics of the ESN reservoir states under perturbations. Figure 9b shows the relative L2 variation in reservoir states between clean and perturbed inputs. The variation remains tightly bound under both Gaussian and die-flip perturbations, without divergent growth, indicating that recurrent state mixing attenuates local disturbances rather than amplifying them. This behavior is consistent with the saturating nonlinearity of the tanh activation in the ESN reservoir, which naturally limits state amplification under perturbations.

At the decision level, we analyze the sensitivity of model outputs by measuring margin drop and logit change between clean and perturbed inputs. As shown in Figure 9c, the hybrid CNN-ESN model exhibits significantly smaller margin degradation and logit variation than the CNN baseline under identical perturbations. This implies that the decision boundary of the hybrid model is less sensitive to input noise.

Taken together, these results demonstrate that ESN-based recurrent aggregation improves robustness by maintaining bounded internal state dynamics and reducing decision-level sensitivity, rather than by altering CNN feature extraction or training strength.

#### 4.5.3. Ablation Study on the Source of Robustness Gain

To further clarify the source of this robustness improvement, an ablation study was conducted under the same CT-NT condition with a high noise level (σ = 0.10), as summarized in Table 6. Four model variants were evaluated: a CNN-only baseline, an ESN-only model, a hybrid CNN-ESN model using a single-direction scanline sequence, and a hybrid CNN-ESN model using a bidirectional scanline sequence. All variants shared the same CNN backbone, training procedure, and dataset split, and differed only in the inclusion of the ESN module and the configuration of the scanline-based sequence encoding.

The ablation results provide further insight into the origin of the robustness gain observed under noisy conditions. The ESN-only model exhibits limited robustness, indicating that the ESN module alone is insufficient to achieve stable performance in the CT-NT scenario. In contrast, integrating scanline-based sequential representations with CNN feature maps leads to a clear improvement in noise robustness, highlighting the importance of combining spatial feature extraction with sequential aggregation. Moreover, the bidirectional scanline configuration consistently outperforms the single-direction variant, suggesting that incorporating multiple traversal directions helps reduce directional bias and improves the stability of spatial feature aggregation.

These results confirm that the reported robustness gain, reaching up to a 9.71 pp improvement over the CNN baseline, primarily arises from the joint effect of CNN-based spatial feature encoding and multidirectional scanline-based ESN aggregation, rather than from the ESN module or CNN backbone in isolation.

#### 4.5.4. Training Stability near Critical Spectral Radius

**Figure 10:**
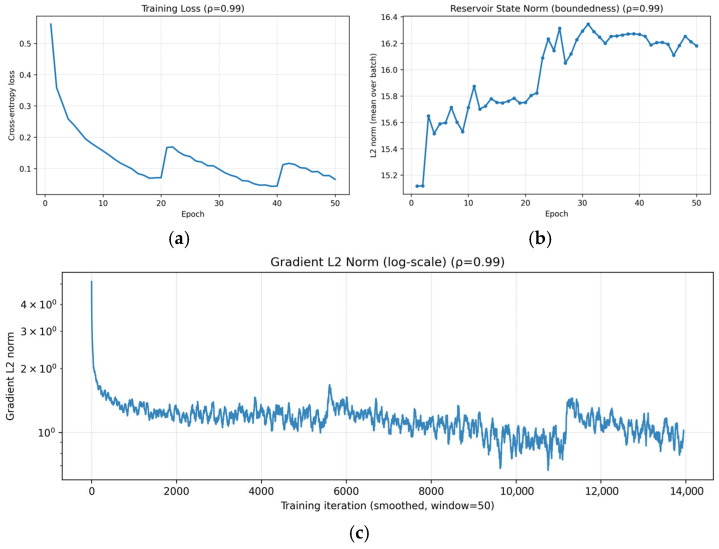
Training stability of the proposed hybrid CNN-ESN model with spectral radius ρ = 0.99: (**a**) training loss over epochs showing stable convergence; (**b**) L2 norm of ESN reservoir states remaining bounded throughout training; (**c**) gradient.

To verify training stability under a near-critical spectral radius, we analyze the learning dynamics of the proposed hybrid CNN-ESN model with ρ = 0.99 using three indicators: training loss, reservoir state norm, and gradient L2 norm. The training loss in Figure 10a decreases without divergence or sustained oscillations, demonstrating stable convergence even when the spectral radius is set close to unity. The mild step-like increases observed around specific epochs correspond to cosine warm-restart learning rate scheduling and mini-batch stochastic optimization effects, rather than instability in the recurrent dynamics. The boundedness of the ESN dynamics is examined by tracking the L2 norm of the reservoir states. As shown in Figure 10b, the reservoir state norm increases during early training and then saturates within a finite range, without unbounded growth. This empirical behavior suggests that the reservoir dynamics remain bounded under the condition ρ < 1, even when the spectral radius is set close to unity. Quantitatively, the reservoir-state L2 norm remains tightly bounded within approximately [15.12, 16.34] across epochs. Numerical stability of optimization is further assessed by the gradient L2 norm in Figure 10c. The gradient L2 norm exhibits a brief transient spike at the first iteration (maximum 31.41), but remains well controlled thereafter (maximum 7.84 after iteration 10; 99% of values below 2.69), with no indication of gradient explosion or vanishing.

Taken together, these results provide empirical evidence of stable and bounded optimization dynamics when ρ = 0.99. Neither reservoir states nor gradients exhibit signs of instability, suggesting that the hybrid CNN-ESN architecture maintains numerically stable training behavior when the spectral radius is set close to unity.

### 4.6. Comparison with Alternative Sequential Models and Inference Efficiency

To contextualize the robustness–efficiency trade-off of the proposed hybrid CNN-ESN architecture, we compare it with two alternative sequence modeling baselines, CNN-LSTM and CNN-Transformer, which are widely used to aggregate ordered feature sequences in vision tasks. The objective of this comparison is not to claim superior computational efficiency, but to assess whether ESN-based sequential aggregation provides a distinctive robustness advantage under test-time perturbations.

All models are trained exclusively on clean data and evaluated under the same CT-NT setting. For the CNN baseline and the proposed hybrid CNN-ESN model, we adopt the paper-exact ResNet34 backbone (conv1-layer4) together with an identical training protocol. For the CNN-LSTM and CNN-Transformer baselines, lightweight CNN-sequence hybrids are constructed by extracting intermediate feature maps from the ResNet34 stem through layer2 and aggregating the resulting spatial token sequences using either (i) a single-layer LSTM with 256 hidden units or (ii) a lightweight Transformer encoder consisting of two encoder layers. This design follows common practice for CNN-sequence hybrids and enables a meaningful robustness comparison against representative sequential modules under the same dataset split and optimization settings.

Table 7 summarizes the comparison results in terms of model complexity, inference latency, and robustness under Gaussian perturbation (σ = 0.10) and binary-consistent die-flip perturbation (*p* = 0.01). Both CNN-LSTM and CNN-Transformer improve robustness over the CNN baseline, but exhibit different robustness characteristics depending on the perturbation type.

Under Gaussian perturbation with σ = 0.10, the proposed hybrid CNN-ESN model achieves the highest accuracy of 87.30%, outperforming the CNN-Transformer (83.99%) by 3.31 percentage points and the CNN-LSTM (79.96%) by 7.34 percentage points. This result suggests that ESN-based sequential aggregation is particularly effective at stabilizing representations under continuous-valued, representation-level distortions.

Under binary-consistent die-flip perturbation, the CNN-Transformer achieves the highest accuracy (81.87%), likely due to its token-level self-attention mechanism, which is well-suited for handling sparse and localized disruptions. The proposed CNN-ESN model remains competitive (79.81%) and continues to outperform the CNN baseline, but does not dominate all alternative sequential models under this perturbation type. These results suggest that different sequential architectures exhibit complementary robustness profiles depending on the nature of the perturbation.

From an efficiency perspective, the CNN-LSTM and CNN-Transformer baselines require fewer parameters and exhibit lower inference latency than the proposed hybrid CNN-ESN model. This is primarily because they operate on earlier-stage CNN feature maps and benefit from highly optimized parallel implementations, particularly in the case of the Transformer. In contrast, the ESN branch performs sequential state updates during scanline-based aggregation, which leads to increased inference latency despite moderate FLOPs. Importantly, the ESN aggregation does not rely on backpropagation through time and introduces no trainable recurrent weights, resulting in stable training dynamics and predictable inference behavior. Overall, these results highlight a clear trade-off: the proposed hybrid CNN-ESN model prioritizes robustness under unseen perturbations at the cost of higher inference latency and parameter count.

### 4.7. Comparison with Robust CNN Training Strategies

To verify that the observed robustness gain is not merely attributable to training-time regularization effects applied to the CNN baseline, we compare the proposed model with common noise-robust CNN training methods, including label smoothing, mixup, and focal loss. All methods are trained on the same clean training set and evaluated on the same noisy test set for a fair comparison.

Under the same CT-NT setting (train on clean data, test on noisy data with σ = 0.10), these methods partially mitigate the degradation of the CNN baseline, reaching 81.09–84.09% accuracy. However, the proposed hybrid CNN-ESN model achieves 87.30%, outperforming all CNN variants under identical training and testing conditions. As summarized in Table 8, even the best-performing robust training method (CNN + Mixup, 84.09%) remains 3.21 percentage points lower than the proposed hybrid CNN-ESN model. Moreover, compared to the plain CNN baseline (77.59%), the hybrid model provides a total improvement of 9.71 percentage points.

This result indicates that the robustness gain cannot be explained solely by training-time regularization or data augmentation. Instead, the architectural integration of CNN-based spatial features with ESN-based recurrent dynamics provides additional stability under test-time noise beyond that obtained by robust CNN training alone in this experimental setting.

### 4.8. Effect of Flattening Strategy in the ESN Branch

To clarify how the sequence construction method affects performance and robustness, we compare two alternative flattening strategies for generating the ESN input sequence: spatial flattening and channel flattening. In spatial flattening, the CNN feature map is reshaped into a sequence along spatial positions (H × W), so that adjacent elements in the sequence correspond to neighboring locations on the wafer. This preserves spatial continuity of defect patterns and allows the ESN to exploit ordered spatial dependencies. In contrast, channel flattening constructs the sequence along the channel dimension, which mixes spatial locations and does not preserve explicit spatial adjacency.

As summarized in Table 9, spatial flattening achieves higher accuracy on clean test data (93.73% vs. 92.84%), indicating that preserving spatial structure is beneficial for discriminative classification. However, under the noisy CT-NT setting, channel flattening shows higher accuracy (90.13% vs. 86.05%) and a much smaller performance drop (2.72 pp vs. 7.68 pp), suggesting stronger noise tolerance due to feature-level smoothing.

These results reveal a trade-off between spatial discrimination and noise robustness. Spatial flattening preserves the ordered spatial progression of wafer defect patterns and therefore robustly captures directional and structural defect layouts, leading to higher accuracy under clean conditions. Channel flattening, on the other hand, reduces sensitivity to perturbations through feature-level smoothing but sacrifices explicit spatial structure. In this work, we adopt spatial flattening as the default design because wafer-map defect classification fundamentally relies on preserving spatial layout and directional structure, while robustness to noise is instead provided by the ESN-based recurrent aggregation and adaptive fusion. While channel flattening exhibits stronger robustness under severe noise, it sacrifices explicit spatial continuity, which is critical for interpreting structured and directional wafer defect patterns.

### 4.9. Results for the NT-CT and NT-NT Scenarios

For the hybrid CNN-ESN model, we additionally report results under noise-injected training conditions to examine how training-time perturbations affect robustness and generalization.

#### 4.9.1. NT-CT

For σ ≤0.10, the difference between model test accuracies in the NT-CT and CT-CT scenarios remained mostly within 0.1–0.3 pp, with no clear trend of improvement or degradation observed. This indicated that the regularization effect of noise injection during training does not substantially improve generalization performance when evaluation is conducted on clean test data. Detailed results are presented in Table A2 (Appendix A).

This behavior can be interpreted in terms of the role of noise-aware training, which is primarily designed to improve robustness under noisy operating conditions rather than to enhance discriminability in noise-free environments. Under the NT-CT settings, representations adapted to accommodate perturbations during training may not translate into improved performance on clean data, reflecting a robustness-discriminability trade-off. Therefore, the limited performance change observed in the NT-CT scenario suggests that the robustness of the proposed hybrid CNN-ESN architecture arises mainly from the inherent structural properties of the ESN branch such as nonlinear saturation, recurrent state mixing, and scanline-based sequential aggregation rather than from noise-based regularization during training.

#### 4.9.2. NT-NT

For σ=0.10, in the NT-NT scenario, the model achieved an accuracy of 94.75% when noise was injected at the feature map stage, whereas in the CT-NT scenario, the model’s accuracy was lower by 7.45 percentage points, reaching 87.30% (Table A3; Appendix A). This suggests that when noise is injected during training and testing, a model adaptation effect occurs.

These results indicate that the proposed hybrid architecture already exhibits a degree of noise robustness arising from the reservoir’s nonlinear saturation and multidirectional averaging structure, even without explicit noise tuning during training. This observation does not imply that noise-aware training is unnecessary; rather, it indicates that the proposed architecture already provides a strong inductive bias for robustness even in the absence of explicit noise tuning.

## 5. Conclusions

In this study, we developed a hybrid CNN-ESN architecture that converts the CNN feature map into a multidirectional scanline sequence and processes it with an ESN to complement conventional spatial feature extraction to address two structural limitations of wafer-map defect classification models based on CNNs: excessive dependence on static spatial patterns and limited robustness under test-time perturbations. The proposed model was designed to adjust the ESN contribution according to the pattern of each defect type and included an adaptive fusion strategy that reflected the ordered spatial dependency of each class.

In the CT-CT scenario, the model achieved high accuracy under clean-data conditions, showing performance improved by 0.61 pp over the baseline CNN. More importantly, the model exhibited substantial robustness improvements over the baseline in the CT-NT scenario, where perturbations were introduced only at evaluation time. Even under high-intensity noise (σ = 0.10), the hybrid model maintained 87.30% accuracy, achieving an absolute performance gain of 9.71 pp over the baseline CNN. This represents an approximately 15.9-fold increase relative to the performance gap observed in the clean-data environment, indicating enhanced robustness to unseen test-time variability rather than gains attributable to noise-aware training.

Per-class analysis showed that the ESN-based sequential representation was particularly beneficial for defect types with clear structural patterns, such as Loc, Edge-Ring, and Edge-Loc, suggesting that the adaptive fusion mechanism effectively leveraged class-dependent spatial organization. For defect types such as Donut, where CNN predictions were already stable, the hybrid model preserved per-class performance while reducing class confusion through balanced fusion.

However, for irregular defect patterns such as Scratch and Near-full, the advantages of the hybrid model were limited or, in some cases, reversed. Scratch defects are sparse and highly localized, and the scanline-to-sequence encoding can smooth such thin patterns through sequential aggregation, reducing their discriminative saliency. Near-full defects contain limited structural variation, resulting in nearly constant scanline sequences, which provide little additional information for the ESN to exploit. Consequently, the ESN-based aggregation process may offer limited benefit or introduce spurious dynamics. These observations indicate an inherent trade-off of ESN-based sequential aggregation: it enhances robustness for structured defect patterns but is less effective for sparse, irregular, or low-information patterns.

Overall, the proposed hybrid CNN-ESN model maintained comparable or higher accuracy under clean-data conditions while improving robustness under noisy test-time evaluation scenarios, suggesting its potential applicability as a robustness-oriented architectural framework rather than a physical process noise model. Future research could further extend the scanline formulation to four directions, explore trainable reservoir dynamics, and develop defect-aware representations and targeted training strategies for irregular defect classes.

## Figures and Tables

**Table 1:** Performance Comparison on WM-811K Dataset.

Method	Year	Backbone	Test Acc (%)	Noise Eval (σ = 0.1)
CNN-WDI [18]	2020	Custom CNN	96.20	-
DCNN [19]	2021	DCNN	93.80	-
DCNN + ECOC-SVM [20]	2021	Custom CNN + SVM	96.40	-
ShuffleNet-v2-CNN [21]	2022	ShuffleNetV2	96.90	-
WM-PeleeNet [22]	2023	LightweightCNN	95.40	-
MFFP-Net [23]	2023	Multi-scale CNN	96.71	-
Autoencode-CNN [24]	2024	AE + CNN	98.56	-
G2LGAN + CNN [13]	2025	GAN + CNN	98.39	-
CNN(Ours)	2025	ResNet34	94.13 ± 0.25	77.59
Hybrid CNN + ESN (Ours)	2025	ResNet34 + ESN	94.74 ± 0.24	87.30 (+9.71 pp)

Note: Previous studies only evaluated test accuracy under clean-data conditions and did not report validation details. This study used a 70/15/15 stratified split and reports the mean performance result for five random seeds.

**Table 2:** Overall Performance in CT-CT Scenario.

Architecture	Acc (%)	Improvement (%)	*p*-Value	Cohen’s d
CNN baseline	94.13 ± 0.25	-	-	-
Hybrid CNN-ESN	94.74 ± 0.24	+ 0.61	0.036	2.49

**Table 3:** Per-Class Accuracy in CT-CT Scenario.

Defect Class	Sample (Test)	CNN Baseline Acc (%)	Hybrid CNN-ESN Acc (%)	Improvement (%)
Center	664	93.55	94.79	+1.24
Donut	83	91.57	91.57	0
Edge-Loc	779	82.70	83.86	+1.16
Edge-Ring	1452	97.18	97.86	+0.21
Loc	539	85.34	87.20	+1.86
Random	130	89.67	89.67	0
Scratch	179	93.93	94.49	+0.56
Near-full	22	95.78	100.00	+4.22

**Table 4:** CT-NT Robustness under Binary Die-flip Perturbation.

Flip Probability (p)	CNN Baseline Acc (%)	Hybrid CNN + ESN Acc (%)	Accuracy Gap (pp)
0.000 (Clean)	94.13	94.74	+0.61
0.001	80.69	82.03	+1.34
0.005	79.65	81.32	+1.67
0.010	77.72	79.81	+2.09
0.020	73.59	75.86	+2.27

Note: Binary-consistent die-flip perturbation preserves the discrete nature of wafer map data and serves as a controlled benchmark for decision-level uncertainty.

**Table 5:** Noise Robustness Comparison in CT-NT Scenario.

Noise Level (σ)	CNN Baseline Acc (%) (Noise)	Hybrid CNN-ESN Acc (%)	Accuracy Gap (%)
0.00 (Clean)	94.13	94.74	+0.61
0.05	92.84 (−1.29 pp)	93.57 (−1.17 pp)	+0.73
0.08	88.85 (−5.28 pp)	91.12 (−3.62 pp)	+2.27
0.10	77.59 (−16.54 pp)	87.30 (−7.44 pp)	+9.71

Note: Robustness under additive Gaussian perturbation in the CT-NT setting. Gaussian noise is used as a representation-level stress test and does not correspond to a physically realistic wafer inspection noise model.

**Table 6:** Ablation Study results (σ = 0.1).

Architecture	Clean (%)	σ = 0.10 (%)	Drop (pp)
CNN baseline	94.13	77.59	16.54
ESN-only	83.56	77.45	6.11
Hybrid CNN + ESN (single direction)	93.86	85.65	8.21
Hybrid CNN + ESN (bi-direction)	94.74	87.30	7.44

Note: Each model was trained and evaluated using multiple independent runs to ensure stable and reliable performance comparison under noisy conditions.

**Table 7:** Robustness–Efficiency Comparison with Alternative Sequential Models (CT-NT).

Model	Params (M)	FLOPs (G)	Latency (ms)	Gaussian (σ = 0.10)	Die-Flip (*p* = 0.01)
CNN baseline	21.44	2.35	3.03	77.59	77.72
CNN + LSTM	2.03	0.34	2.16	79.96	78.97
CNN + Transformer	2.50	0.34	1.87	83.99	81.87
Hybrid CNN + ESN (Ours)	22.37	0.60	4.93	87.30	79.81

**Table 8:** Accuracy comparison with noise-robust CNN training methods (CT-NT, σ = 0.10).

Method	Accuracy (%)	Baseline (pp)	Hybrid CNN-ESN (pp)
CNN baseline	77.59	-	−9.71
CNN + Label Smoothing	81.09	+3.50	−6.21
CNN + Focal Loss	82.26	+4.67	−5.04
CNN + Mixup	84.09	+6.50	−3.21
Hybrid CNN + ESN (Ours)	87.30	+9.71	-

**Table 9:** Effect of flattening strategy on accuracy and robustness.

Flattening Strategy	Clean Acc (%)	CT-NT Acc (%)	Accuracy Drop (pp)
Spatial flatten (Ours)	93.73	86.05	7.68
Channel flatten	92.84	90.13	2.72

**Figure A1:**
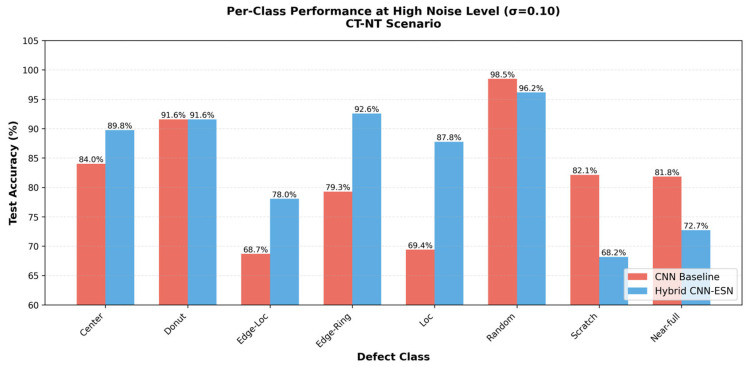
Per-class accuracy comparison under high noise conditions (σ = 0.10) in the CT-NT scenario.

## Data Availability

The data presented in this study are available on request from the corresponding author.

## Appendix A. Supplementary Experimental Details

### Appendix A.1. Class-Specific Adaptive Fusion Weights

Table A1 shows the optimized ESN contribution weights ($\omega_{c}$) for each defect class. We performed a grid search with $\omega_{c}$ $\in \left[0,\;1\right]$ at 0.05 intervals, and the value with the highest mean validation accuracy across five random seeds was selected as the final weight for each class.

**Table A1:** Class-wise Adaptive Fusion Weights ($\omega_{c}$).

Defect Class	α (ESN Contribution)	Rationale
Center	0.05	Static pattern fixed at the center; almost no sequential progression
Donut	0.50	Radial ring structure exhibiting directionality and progression
Edge-Loc	0.35	Localized edge progression patterns; moderate sequential dependency
Edge-Ring	0.30	Continuous edge sequence with limited progression
Loc	0.40	Localized spreading pattern with a partial sequential structure
Random	0.00	No structural order; ESN contributions removed
Scratch	0.60	Linear progression pattern; strong sequence dependency
Near-full	0.10	Global spread; low sequential dependency

Note: A higher $\omega_{c}$ value indicates a larger ESN contribution. Classes with structural progression tendencies were assigned higher weights.

### Appendix A.2. NT-CT Analysis by Injection Position

Table A2 presents the results for the NT-CT scenario across various noise levels and injection positions.

**Table A2:** NT-CT Accuracy (%) by Noise Level (σ) and Injection Position.

Noise σ	Input	Feature Map	ESN Input	Fusion
0.001	94.41	94.45	94.47	94.46
0.005	94.54	94.46	94.45	94.45
0.01	94.57	94.53	94.56	94.57
0.03	94.40	94.57	94.48	94.73
0.05	94.47	94.42	94.48	94.39
0.08	92.14	94.65	94.50	94.49
0.10	91.73	94.75	94.53	94.52

When noise was injected in input, accuracy dropped sharply to <92% for σ ≥ 0.08. Meanwhile, when noise was injected in the feature map, ESN input, and fusion layer, accuracies were in the 94.4–94.8% range across all noise levels.

**Table A3:** Comparison of CT-NT and NT-NT Accuracy at σ =0.10.

Training Condition	Noise Injection Position	Acc (%)	Improvement over CT-NT
Clean Training	Input	87.30	-
Noise Training	Input	91.73	+4.43
Noise Training	Feature Map	94.75	+7.45
Noise Training	ESN Input	94.53	+7.23
Noise Training	Fusion	94.52	+7.22

Note: The CT-NT scenario reflects evaluation conditions in which noise is introduced only at the testing stage. In the NT-NT scenario, the model showed an adaptation effect when trained and tested under matching noise conditions, achieving 94.75% accuracy at σ = 0.10, substantially higher than the 87.30% observed in the CT-NT scenario. Noise injection in the feature map led to the highest model robustness.

### Appendix A.3. Per-Class Noise Robustness Analysis

Figure A1 illustrates the trends of per-class performance degradation. For σ = 0.10, the CNN baseline showed steep accuracy drops across most classes, whereas the hybrid model maintained higher accuracy for classes with clear structural patterns. The accuracy gap between the two models became even more pronounced for high-intensity noise.

Per-class analysis further reveals that the robustness gain of the proposed hybrid model is class-dependent. Under σ = 0.10, the hybrid CNN-ESN substantially outperforms the CNN baseline for defect classes with clear and repetitive structural patterns, such as Loc, Edge-Ring, and Center.

In contrast, performance degrades for irregular and weakly structured defects such as Scratch, where the hybrid model underperforms the CNN baseline.

This observation suggests that ESN-based sequential aggregation is most effective for structured spatial patterns, while sparse and fragmented defects may not benefit from scanline-based smoothing.

**Table A4:** Per-Class Noise Robustness at σ =0.1.

Defect Class	CNN Baseline Acc (%) (Noise)	Hybrid CNN-ESN Acc (%) (Noise)	Hybrid Advantage (%)
Center	84.01	89.75	+5.74
Edge-Loc	68.68	78.05	+9.37
Edge-Ring	79.27	92.56	+13.29
Loc	69.39	87.76	+18.37
Scratch	82.12	68.16	−13.96

**Table A5:** Full Per-Class Noise Robustness at σ = 0.10.

Defect Class	CNN Baseline Acc (%) (Noise)	Hybrid CNN-ESN Acc (%) (Noise)	Hybrid Advantage (%)
Center	84.01	89.75	+5.74
Donut	91.57	91.57	0.00
Edge-Loc	68.68	78.05	+9.37
Edge-Ring	79.27	92.56	+13.29
Loc	69.39	87.76	+18.37
Random	98.46	96.15	−2.31
Scratch	82.12	68.16	−13.96
Near-full	81.82	72.73	−9.09

Note: The accuracy difference between the two models was determined under the same noise condition. For Scratch, Near-full, and Random, the hybrid model showed limited or lower performance compared to the CNN baseline.
